# Association between exon-3 polymorphism of the GH Receptor(GHR) gene with catch growth in children born small for gestational age

**DOI:** 10.1186/1687-9856-2013-S1-P207

**Published:** 2013-10-03

**Authors:** Il Tae Hwang, Shim Young Shim

**Affiliations:** 1 Department Of Pediatrics, Kangdong Sacred Heart Hospital, Hallym Univertsity, College Of Medicine

## Objective

The aim of this study was to investigate relationship between GHR exon 3 genotype and catch up growth in children fullterm born small for gestational age.

## Methods

Children were classified as SGA if birth weight was less than -2SDS. Catch up defined as height of less than -2SDS at the time the child was examined. DNA for the GHR exon 3 genotyping was isolated from peripheral blood lymphocytes and was analyzed by multiplex PCR.

## Results

1) Total of 92 children, 74(80.4%) had sufficiently catch up. 2) 69(75%) were *fl/fl*, 15(16.3%) were *fl/d3* and 8(8.6%) were *d3/d3* the SGA groups, compared with 81%, 18%, and 1%, respectively in the Korean controls(*P*=0.04). 3) The highest catch up rate was seen in the fl/d3 subgroup(86.6%), the lowest in the d3/d3 subgroup (62.5%). The differences were not significant. 3)*d3/d3* GHR genotype in the SGA groups is higher than in the Korean controls. 4) Fl/fl GHR genotype is higher than other published data in SGA groups.

## Conclusion

Differences in the frequency distribution of the GHR polymorphism genotype between normal Korean population and SGA groups were not significant. GHR-exon 3 polymorphism did not influence the postnatal growth in Korean children with SGA. Additional studies are required to establish effect of the d3-GHR allele on prenatal growth in SGA.

